# Single cell PCR amplification of diatoms using fresh and preserved samples

**DOI:** 10.3389/fmicb.2015.01084

**Published:** 2015-10-14

**Authors:** Paul B. Hamilton, Keely E. Lefebvre, Roger D. Bull

**Affiliations:** ^1^Research and Collections, Canadian Museum of NatureOttawa, ON, Canada; ^2^Biology and Environmental Science, Center for Advanced Research in Environmental Genomics, University of OttawaOttawa, ON, Canada

**Keywords:** single cells, phylogenetics, diatoms, RNA*later*, fixation, barcoding

## Abstract

Single cell Chelex® DNA extraction and nested PCR amplification were used to examine partial gene sequences from natural diatom populations for taxonomic and phylogenetic studies at and above the level of species. DNA was extracted from cells that were either fresh collected or stored in RNA*later*. Extractions from Lugol's fixation were also attempted with limited success. Three partial gene sequences (rbcL, 18S, and psbA) were recovered using existing and new primers with a nested or double nested PCR approach with amplification and success rates between 70 and 96%. An rbcL consensus tree grouped morphologically similar specimens and was consistent across the two primary sample treatments: fresh and RNA*later*. This tool will greatly enhance the number of microscopic diatom taxa (and potentially other microbes) available for barcoding and phylogenetic studies. The near-term increase in sequence data for diatoms generated via routine single cell extractions and PCR will act as a multiproxy validation of longer-term next generation genomics.

## Introduction

DNA barcoding has become common practice in animal and plant taxonomy (Hebert et al., 2003) with cytochrome c oxidase 1 (CO1), a mitochondrial gene, serving as the main animal barcoding gene (Hebert et al., 2004). In plants the chloroplast genes ribulose 1,5-biphosphate (rbcL) and megakaryocyte-associated tyrosine kinase (MatK) serve as two of the preferred barcode genes for taxonomic identifications (CBOL Plant Work Group, 2009). This is in contrast with the situation faced in diatom barcoding where several regions are presently identified as prominent taxonomic markers (e.g., Yoon et al., 2002; Evans et al., 2007). In some studies the conservative rbcL, CO1, and ribosomal complex gene 18S are considered to be good taxonomic characters for species determinations (Mann et al., 2010; Hamsher et al., 2011; Zimmermann et al., 2011). Ribosomal complex genes ITS, 18S, and 28S were also used both individually and in multi-gene studies to evaluate cryptic taxonomic variations at the genus and species levels (e.g., Amato et al., 2007; Vanormelingen et al., 2008; Pouličková et al., 2010; Kaczmarska et al., 2014). The smaller (~500 bp) chloroplast psbA gene was used in evolutionary studies but is not variable enough to be informative for species-level taxonomic studies (Souffreau et al., 2011, pers. obs.). In contrast, chloroplast gene psbC (>1000 bp) is more widely used across all the major orders (Theriot et al., 2010). With little consensus as to which marker best delimits diatom groups, the ability to amplify several genes including new genes from a single cell is essential for diatom taxonomy using DNA barcodes.

In microbial studies, genetic sequencing has been successful across all the primary algal families Bacillariophyceae, Cyanophyceae, Chlorophyceae, Chrysophyceae, Cryptophyceae, Desmidiaceae, Euglenophyceae, Haptophyceae, Pyrrhophyceae, Raphidophyceae, Synurophyceae (e.g., Daugbjerg and Andersen, 1997; McCourt et al., 2000; Tomitani et al., 2006; Edvardsen et al., 2011; Bennett et al., 2014). However, compared to DNA research in Plantae and Animalae, there are much fewer sequences available for diatom taxa, leaving large taxonomic holes in DNA databases. As well, microbial genetic studies in algae are limited by the ability to collect enough material, and population genetic studies are all but absent. Culturing algae and other microbes to accumulate sufficient DNA has been the time-limiting step in microbial genetics. Cultures supply extra material for morphological identification and validation; however cultures are prone to alterations in structural morphology (Trainor et al., 1971; Estes and Dute, 1994).

Single cell extraction and PCR protocols have been advanced in microbe research on live and fixed materials, although no single approach meets all aspects of routine enhanced multi-gene taxonomic research (e.g., Sherbakova et al., 2000; Ruiz Sebastián and O'Ryan, 2001; Lang and Kaczmarska, 2011). To date single cell extractions have been successfully completed with the Chrysophyceae (Auinger et al., 2008), Pyrrhophyceae (Richlen and Barber, 2005; Henrichs et al., 2007), and Bacillariophyceae (Lang and Kaczmarska, 2011). There are only a few published examples using non-cultured single cell amplifications (e.g., Godhe et al., 2002; Auinger et al., 2008; Lang and Kaczmarska, 2011). These amplifications are successful if there is sufficient initial DNA within the cell and the primer set is effective at maximizing amplification efficiency. In most cases, amplification of DNA for sequencing requires a nested amplification protocol. This nested approach is effective but has the potential to generate amplification errors (Ruck et al., 2014). In order to effectively utilize this nested approach, clear protocols for error checking and sequence validation much be established. Single cell genetic determinations are presently a novel and potentially efficient way to generate a large reference library of taxonomically informative data from single algal cells in complex environmental systems. This reference library will also contain an extensive database for population genetics and genetic biogeography studies (e.g., Alverson et al., 2007).

There are a number of reagents which can be used in single cell DNA amplifications with algae (e.g., Bertozzini et al., 2005; Auinger et al., 2008). Chelex® resin is an effective DNA extraction tool with applications in molecular biology ranging from multicellular vertebrate tissues to single microbial cells (Hahn et al., 2000; Richlen and Barber, 2005). This extraction method was used in genetic investigations in forensic science (Legrand et al., 2002), population studies (Richlen and Barber, 2005), and evolution (Theriot et al., 2010). In mammalian research Chelex extractions have been used on fresh, frozen, alcohol preserved, and to a limited degree on formalin-fixed tissues (Legrand et al., 2002). The simplicity of this technique coupled with relatively cheap cost allows for quick PCR assays (e.g., Bowers et al., 2000; Reyes-Escogido et al., 2010) which has the potential for more detailed taxonomic barcoding initiatives and finer population genetic studies.

The recovery of gene sequences from structurally fixed and preserved material has had some success across all the biological groups, but has not developed into a routine protocol used for sequencing (Connell, 2002; Godhe et al., 2002; Henrichs et al., 2007). Institutions with collections of fixed biological materials, like museums, are extremely interested in the recovery of genes and genomes from their historic collections. The temporal record hidden in fixed biological collections—with regard to speciation and population genetics—is waiting to be mined. In microbial genetics, gene sequences have been recovered from ethanol, Lugol's solution, buffered formalin and RNA*later* fixations (Ambion, 1999; Connell, 2002; Godhe et al., 2002; Auinger et al., 2008; this study). The general recipe for success is removal or dilution of the traditional fixation solution followed by standard sample processing, DNA extraction, and gene sequencing. Buffered formalin preserved materials can be treated with cold methanol to minimize the impact of the fixation, while sodium thiosulfate is effective in capturing iodine from solutions and biological materials (Godhe et al., 2002; Auinger et al., 2008). In the Protista, ethanol preparation and fixation prior to sequencing is less common, although has been successful with some limitations (e.g., Godhe et al., 2002; Henrichs et al., 2007; Lang and Kaczmarska, 2011; Ivanova et al., submitted). RNA*later* is a more recent stabilizer suitable for the storage of material prior to RNA and DNA sequencing with a high rate of recovery (Ambion, 1999). RNA*later* has the advantage of being a good fixative and further prevents the degradation of RNA and DNA. At 4°C viable fixation can be maintained for a month and at −20°C samples can be maintained indefinitely (Ambion, 1999).

The objective of this study was to evaluate a nested amplification protocol for multiple genes in diatoms from single cells under live and RNA*later* preserved conditions. This will establish a standard multiproxy routine for reproducible barcoding with morphological analysis of natural populations. In this study new primer pairs for nested amplification of rbcL, 18S and psbA were designed and different DNA polymerases and cycling protocols were compared. Finally, examples are presented for how this protocol can be used to establish a more comprehensive reference library of taxonomic and physiological genetic data.

## Materials and methods

### Collection of samples

All samples were benthic or planktonic and collected from freshwaters with a wide range in pH (5.1–7.8) and eutrophication states (oligotrophic–mesotrophic) (Tables 1, 2). The samples were kept cool in transport and at room temperature in the lab in natural light prior to single cell isolations.

**Table 1:** **List of 35 taxa isolated and sequenced for genes rbcL, psbA, and 18S, with Canadian Museum of Nature Accession numbers, GenBank Id numbers, sample media, source locality with date of collection, and collector**.

**Taxon**	**Accession number**	**GenBank ID for rbcL**	**GenBank ID for 18S**	**GenBank ID for psbA**	**Identifier**	**Sample medium**	**Locality and date of collection**	**Collector**
*Stauroneis* cf*. gracilis*	CANA 93655	KM999045	KM998975	KM999010	A6R6	Water	Small lake near Lac a Serpent (Quebec, Canada) 45.627633 N 75.601467 W, 07-Sep-2013	F.D. Caron
*Stauroneis* cf*. gracilis*	CANA 93655	KM999046	KM998976	KM999011	A5R6	Water	Small lake near Lac a Serpent (Quebec, Canada) 45.627633 N 75.601467 W, 07-Sep-2013	F.D. Caron
*Stauroneis* cf*. anceps*	CANA 93655	KM999047	KM998977	KM999012	B3R6	Water	Small lake near Lac a Serpent (Quebec, Canada) 45.627633 N 75.601467 W, 07-Sep-2013	F.D. Caron
*Pinnularia* sp.	CANA 93252	KM999048	KM998978	KM999013	B5R10	RNALater	Alymer, small pond (Quebec, Canada) 45.443007 N 75.810437 W, 30-Jan-2014	P.B. Hamilton
*Pinnularia* sp.	CANA 93252	KM999049	KM998979	KM999014	A2R10	Water	Alymer, small pond (Quebec, Canada) 45.443007 N 75.810437 W, 30-Jan-2014	P.B. Hamilton
*Pinnularia* sp.	CANA 93251	KM999050	KM998980	KM999015	A1R10	Water	Alymer, small pond (Ontario, Canada) 45.442997 N 75.810437 W, 30-Jan-2014	P.B. Hamilton
*Pinnularia* sp.	CANA 93250	KM999051	KM998981	KM999016	A3R10	Water	Alymer, small pond (Quebec, Canada) 54.443033 N 75.810378 W, 30-Jan-2014	P.B. Hamilton
*Pinnularia* sp.	CANA 93249	KM999052	KM998982	KM999017	A4R10	Water	Alymer, small pond (Quebec, Canada) 45.443022 N 75.810421 W, 30-Jan-2014	P.B. Hamilton
*Pinnularia* sp.	CANA 93251	KM999053	KM998983	KM999018	B1R10	RNALater	Alymer, small pond (Quebec, Canada) 45.442997 N 75.810437 W, 30-Jan-2014	P.B. Hamilton
*Pinnularia* sp.	CANA 93641	KM999054	KM998984	KM999019	B1R7	RNALater	Ditch, Hwy 56, Adirondack Park (New York, USA) 44.30692 N 74.71650 W, 26-Oct-2013	P.B. Hamilton
*Pinnularia* sp.	CANA 93250	KM999055	KM998985	KM999020	A8R10	RNALater	Alymer, small pond (Quebec, Canada) 54.443033 N 75.810378 W, 30-Jan-2014	P.B. Hamilton
*Pinnularia* sp.	CANA 93660	KM999056	KM998986	KM999021	E3R8	Water	Alymer, small pond (Quebec, Canada) 45.443033 N 75.810383 W, 15-Nov-2013	K.E. Lefebvre
*Navicula* sp.	CANA 93656	KM999057	KM998987	KM999022	B8R6	Water	Small lake near Lac a Serpent (Quebec, Canada) 45.627633 N 75.601467 W, 28-Sep-2013	F.D. Caron
*Navicula* sp.	CANA 93655	KM999058	KM998988	KM999023	B7R6	Water	Small lake near Lac a Serpent (Quebec, Canada) 45.627633 N 75.601467 W, 07-Sep-2013	F.D. Caron
*Navicula* sp.	CANA 93655	KM999059	KM998989	KM999024	B6R6	Water	Small lake near Lac a Serpent (Quebec, Canada) 45.627633 N 75.601467 W, 07-Sep-2013	F.D. Caron
*Navicula* sp.	CANA 93655	KM999060	KM998990	KM999025	B5R6	Water	Small lake near Lac a Serpent (Quebec, Canada) 45.627633 N 75.601467 W, 07-Sep-2013	F.D. Caron
*Navicula* sp.	CANA 93655	KM999061	KM998991	KM999026	B4R6	Water	Small lake near Lac a Serpent (Quebec, Canada) 45.627633 N 75.601467 W, 07-Sep-2013	F.D. Caron
*Navicula* sp.	CANA 93655	KM999062	KM998992	KM999027	B1R6	Water	Small lake near Lac a Serpent (Quebec, Canada) 45.627633 N 75.601467 W, 07-Sep-2013	F.D. Caron
*Navicula* sp.	CANA 93656	KM999063	KM998993	KM999028	C6R6	Water	Small lake near Lac a Serpent (Quebec, Canada) 45.627633 N 75.601467 W, 28-Sep-2013	F.D. Caron
*Navicula* sp.	CANA 93656	KM999064	KM998994	KM999029	C3R6	Water	Small lake near Lac a Serpent (Quebec, Canada) 45.627633 N 75.601467 W, 28-Sep-2013	F.D. Caron
*Sellephora* sp.	CANA 93647	KM999065	KM998995	KM999030	F4R6	Water	Milledgeville, south of airport (Georgia, USA) 33.1472 N 83.2505 W, 22-Oct-2013	P.B. Hamilton
*Craticula* cf. *cuspidata*	CANA 93656	KM999066	KM998996	KM999031	C4R6	Water	Small lake near Lac a Serpent (Quebec, Canada) 45.627633 N 75.601467 W, 28-Sep-2013	F.D. Caron
*Craticula* cf. *cuspidata*	CANA 93655	KM999067	KM998997	KM999032	A3R6	Water	Small lake near Lac a Serpent (Quebec, Canada) 45.627633 N 75.601467 W, 07-Sep-2013	F.D. Caron
*Craticula* cf. *cuspidata*	CANA 93655	KM999068	KM998998	KM999033	A2R6	Water	Small lake near Lac a Serpent (Quebec, Canada) 45.627633 N 75.601467 W, 07-Sep-2013	F.D. Caron
*Craticula* cf. *cuspidata*	CANA 93655	KM999069	KM998999	KM999034	A1R6	Water	Small lake near Lac a Serpent (Quebec, Canada) 45.627633 N 75.601467 W, 07-Sep-2013	F.D. Caron
*Craticula cuspidata*	CANA 93647	KM999070	KM999000	KM999035	C6R7	RNALater	Milledgeville, south of airport (Georgia, USA) 33.1472 N 83.2505 W, 22-Oct-2013	P.B. Hamilton
*Craticula* cf*. cuspidata*	CANA 93656	KM999071	KM999001	KM999036		Water	Small lake near Lac a Serpent (Quebec, Canada) 45.627633 N 75.601467 W, 28-Sep-2013	F.D. Caron
*Gyrosigma accuminatum*	CANA 93251	KM999072	KM999002	KM999037		RNALater	Alymer, small pond (Quebec, Canada) 45.442997 N 75.810437 W, 30-Jan-2014	P.B. Hamilton
*Gyrosigma accuminatum*	CANA 93251	KM999073	KM999003	KM999038		Water	Alymer, small pond (Quebec, Canada) 45.442997 N 75.810437 W, 30-Jan-2014	P.B. Hamilton
*Gyrosigma accuminatum*	CANA 93251	KM999074	KM999004	KM999039		Water	Alymer, small pond (Quebec, Canada) 45.442997 N 75.810437 W, 30-Jan-2014	P.B. Hamilton
*Gyrosigma accuminatum*	CANA 93249	KM999075	KM999005	KM999040		RNALater	Alymer, small pond (Quebec, Canada) 45.443022 N 75.810421 W, 30-Jan-2014	P.B. Hamilton
*Gyrosigma accuminatum*	CANA 93663	KM999076	KM999006	KM999041		RNALater	Alymer, small pond (Quebec, Canada) 45.443050 N 75.810367 W, 15-Nov-2013	K.E. Lefebvre
*Gyrosigma accuminatum*	CANA 93663	KM999077	KM999007	KM999042		RNALater	Alymer, small pond (Quebec, Canada) 45.443050 N 75.810367 W, 15-Nov-2013	K.E. Lefebvre
*Gyrosigma accuminatum*	CANA 93663	KM999078	KM999008	KM999043		RNALater	Alymer, small pond (Quebec, Canada) 45.443050 N 75.810367 W, 15-Nov-2013	K.E. Lefebvre
*Cymatopleura solea*	CANA 93251	KM999079	KM999009	KM999044		RNALater	Alymer, small pond (Quebec, Canada) 45.442997 N 75.810437 W, 30-Jan-2014	P.B. Hamilton

**Table 2:** **List of 38 taxa isolated and sequenced for the gene rbcL, with Canadian Museum of Nature Accession numbers, GenBank Id numbers, sample media, source locality with date of collection, and collector**.

**Taxon**	**Accession number**	**GenBank ID**	**Identifier**	**Sample medium**	**Locality and date of collection**	**Collector**
*Melosira varians*	CANA 100020	KM999080	I7R14	Water	Black Rapids, Rideau River (Ontario, Canada) 45.321990 N 75.699427 W, 19-Jun-2014	P.B. Hamilton
*Melosira varians*	CANA 100020	KM999081	I4R14	Water	Black Rapids, Rideau River (Ontario, Canada) 45.321990 N 75.699427 W, 19-Jun-2014	P.B. Hamilton
*Synedra* sp.	CANA 93622	KM999082	C7R5	Water	Alymer, small pond (Quebec, Canada) 45.442838 N 75.810296 W, 04-Oct-2013	P.B. Hamilton
*Surirella*	CANA 100068	KM999083	A7R14	Water	Petawawa Forestry Station (Ontario, Canada) 45.992966 N 77.399194 W, 18-May-2014	P.B. Hamilton
*Surirella*	CANA 93281	KM999084	B6R13	Water	Creek, Adirondack Park (New York, USA) 44 00.715 N 74 49.155 W, 11-May-2014	P.B. Hamilton
*Cymatopleura solea*	CANA 93636	KM999085	G3R5	Water	Alymer, small pond (Quebec, Canada) 45.442970 N 75.810277 W, 10-Oct-2013	P.B. Hamilton
*Cymatopleura solea*	CANA 93622	KM999086	C2R5	Water	Alymer, small pond (Quebec, Canada) 45.442838 N 75.810296 W, 04-Oct-2013	P.B. Hamilton
*Stauroneis* cf*. gracilis*	CANA 93639	KM999087	B8R5	Water	Ditch, Hwy 36 (Ontario, Canada) 44.803911 W 76.509686 W, 28-Sep-2013	P.B. Hamilton
*Sellephora cf. laevissima*	CANA 100068	KM999088	B2R14	Water	Petawawa Forestry Station (Ontario, Canada) 45.992966 N 77.399194 W, 18-May-2014	P.B. Hamilton
*Neidium* sp.	CANA 100068	KM999089	G3R12	Water	Petawawa Forestry Station (Ontario, Canada) 45.992966 N 77.399194 W, 18-May-2014	P.B. Hamilton
*Neidium tumescens*	CANA 100072	KM999090	C7R12	Water	Wetland, Adirondack Park (New York, USA) 43.90870 N 74.43944 W, 11-May-2014	P.B. Hamilton
*Neidium tumescens*	CANA 100072	KM999091	A7R12	Water	Wetland, Adirondack Park (New York, USA) 43.90870 N 74.43944 W, 11-May-2014	P.B. Hamilton
*Neidium* sp.	CANA 93372	KM999092	D1R12	Water	Pond, Hwy 56 (New York, USA) 44.39536 N 74.75690 W, 11-May-2014	P.B. Hamilton
*Cymbella* sp.	CANA 100068	KM999093	H1R12	Water	Petawawa Forestry Station (Ontario, Canada) 45.992966 N 77.399194 W, 18-May-2014	P.B. Hamilton
*Cymbopleura subcuspidata*	CANA 100068	KM999094	H4R12	Water	Petawawa Forestry Station (Ontario, Canada) 45.992966 N 77.399194 W, 18-May-2014	P.B. Hamilton
*Cymbopleura subcuspidata*	CANA 93374	KM999095	D7R13	Water	Wetland, Adirondack Park (New York, USA) 44.31925 N 74.72392 W, 10-May-2014	P.B. Hamilton
*Cymbopleura subcuspidata*	CANA 93374	KM999096	D6R13	Water	Wetland, Adirondack Park (New York, USA) 44.31925 N 74.72392 W, 10-May-2014	P.B. Hamilton
*Gomphonema cf. parvulum*	CANA 93654	KM999097	A6R8	Water	Alymer, small pond (Quebec, Canada) 45.44288 N 75.81045 W, 05-Nov-2013	K.E. Lefebvre
*Encyonema* sp.	CANA 93281	KM999098	B8R13	Water	Creek, Adirondack Park (New York, USA) 44.00715 N 74.49155 W, 11-May-2014	P.B. Hamilton
*Frustulia bahlsii*	CANA 93284	KM999099	C6R13	Water	Big Moose Lake, Adirondack Park (New York, USA) 43.81723 N 74.88400 W, 10-May-2014	P.B. Hamilton
*Frustulia bahlsii*	CANA 93279	KM999100	A5R13	Water	Wetland, Adirondack Park (New York, USA) 44.44806 N 74.77439 W, 10-May-2014	P.B. Hamilton
*Frustulia bahlsii*	CANA 93284	KM999101	C5R13	Water	Big Moose Lake, Adirondack Park (New York, USA) 43.81723 N 74.88400 W, 10-May-2014	P.B. Hamilton
*Frustulia* cf*. saxonica*	CANA 93281	KM999102	B5R13	Water	Creek, Adirondack Park (New York, USA) 44.00715 W 74.49155 W, 11-May-2014	P.B. Hamilton
*Gyrosigma accuminatum*	CANA 93253	KM999103	E5R10	Iodine	Alymer, small pond (Quebec, Canada) 45.443031 N 75.810467 W, 21-Mar-2014	P.B. Hamilton
*Gyrosigma accuminatum*	CANA 93663	KM999104	G3R9	RNALater	Alymer, small pond (Quebec, Canada) 45.26583 N 75.48622 W, 15-Nov-2013	K.E. Lefebvre
*Navicula* sp.	CANA 93656	KM999105	C5R6	Water	Small lake near Lac A Serpent (Quebec, Canada) 45.627633 N 75.601467 W, 28-Sep-2013	F.D. Caron
*Navicula* sp.	CANA 93656	KM999106	D1R6	Water	Small lake near Lac A Serpent (Quebec, Canada) 45.627633 N 75.601467 W, 28-Sep-2013	F.D. Caron
*Navicula* sp.	CANA 93656	KM999107	C1R6	Water	Small lake near Lac A Serpent (Quebec, Canada) 45.627633 N 75.601467 W, 28-Sep-2013	F.D. Caron
*Navicula* sp.	CANA 93645	KM999108	E5R6	Water	Alymer, small pond (Quebec, Canada) 45.44305 N 75.81043 W, 08-Oct-2013	P.B. Hamilton
*Navicula* sp.	CANA 93645	KM999109	E3R6	Water	Alymer, small pond (Quebec, Canada) 45.44305 N 75.81043 W, 08-Oct-2013	P.B. Hamilton
*Navicula* sp.	CANA 93644	KM999110	D3R6	Water	Alymer, small pond (Quebec, Canada) 45.44305 N 75.81043 W, 08-Oct-2013	P.B. Hamilton
*Navicula* sp.	CANA 93644	KM999111	D5R6	Water	Alymer, small pond (Quebec, Canada) 45.44305 N 75.81043 W, 08-Oct-2013	P.B. Hamilton
*Navicula* cf*. cryptocephala*	CANA 93655	KM999112	A8R6	Water	Small lake near Lac A Serpent (Quebec, Canada) 45 37.658 N 75 36.088 W, 07-Sep-2013	P.B. Hamilton
*Nitzschia* cf*. sigmoidea*	CANA 100068	KM999113	G2R12	Water	Petawawa Forestry Station (Ontario, Canada) 45.992966 N 77.399194 W, 18-May-2014	P.B. Hamilton
*Nitzschia linearis*	CANA 93374	KM999114	D8R13	Water	Adirondack Park (New York, USA) 44.31925 N 74.72392 W, 10-May-2014	P.B. Hamilton
*Eunotia* sp.	CANA 93373	KM999115	D2R13	Water	Big Moose Lake, Adirondack Park (New York, USA) 43.81782 N 74.88404 W, 10-May-2014	P.B. Hamilton
*Aulacoseria granulata*	CANA 100020	KM999116	I6R14	Water	Black Rapids, Rideau River (Ontario, Canada) 45.321990 N 75.699427 W, 19-Jun-2014	P.B. Hamilton
*Aulacoseria granulata*	CANA 100020	KM999117	I3R14	Water	Black Rapids, Rideau River (Ontario, Canada) 45.321990 N 75.699427 W, 19-Jun-2014	P.B. Hamilton

### RNA*later* preservation

A 0.2 mL volume of fresh benthic sample was aliquoted into a 1.5 mL tube containing 1 mL of RNA*later* tissue storage buffer (http://www.protocol-online.org/prot/Protocols/RNAlater-3999.html). The sample was hand shaken to mix, kept at room temperature for 24 h, and then stored in the dark at 4°C between 5 and 21 days before single cell isolation.

### Lugol's fixation

Following the protocol from Henrichs et al. (2007), 0.2 mL of living benthic sample was aliquoted into a 1.5 mL tube and then fixed with Lugol's iodine solution (10 g I_2_, 20 g of KI, 200 mL ddH_2_O). In this study non-acidified Lugol's fixation (no glacial acetic acid) was used while Henrichs et al. (2007) used acidified Lugol's fixation. The merits of using non-acidified vs. acidified Lugol's fixatuion, was discussed in Throndsen (1978). Samples fixed with non-acidified Lugol's iodine solution varied in storage duration from 12 days to 20 years. Just prior to isolation, 20 μL of 1 M sodium thiosulfate was added to the samples and hand mixed until the Lugol's iodine solution was dissipated (became colorless).

### Single diatom cell isolation

One to two milliliters of each sample (living, RNA*later* preserved or Lugol's preserved) was placed on a large microscope slide. The sample was then diluted with ~1 mL of sterile, nuclease free water (Bioshop Canada Inc.) and examined under an inverted microscope (Leica) or a compound microscope (Nikon, with long working distance objectives) at 10x magnification. Individual cells were isolated through suction using 20–40 μl drawn-out disposable pipets, either with a Narishige micromanipulator or simple manual suction. This isolation procedure was modified from Throndsen (1978) by removing the use of Formvar film. The isolated cell with associated contaminants was transferred to a new water droplet of DNA nuclease free water. This isolation and transfer was repeated 2–5 times to remove any contaminants and/or preservative residue. Individual cells were then isolated for the final time and transferred to a 0.2 mL PCR tube containing 200 μL of 10% (w/v) Chelex® 100 solution (Richlen and Barber, 2005). The samples were stored from 1 to 51 days at 4°C in the dark until DNA extraction (Tables 1, 2; Supplement 1 in Appendix).

### DNA extraction, PCR and sequencing protocol

For DNA extraction, Chelex-stored samples were incubated for 20 min at 95°C. They were then vortexed for 15 s and centrifuged for 15 s at 14,000 rcf. We also tested extraction without the incubation step on some samples, and had similar success with both protocols. For PCR study, the following primers were used (Table 3).

**Table 3:** **Oligonucleotide primer sequences used in the nested amplifications**.

**Nested**	**Forward primers: name, (sequence)**	**Reverse primers: name, (sequence)**	**Author**
a	rbcL66+ (5′-TTTAAGGAGAAATAAATGTCTCAATCTG-3′)	rbcLdp7- (5′-AAASHDCCTTGTGTWAGTVTC-3′)	Alverson et al., 2007; Daugbjerg and Andersen, 1997
a	rbcL40+ (5′-GGACTCGAATVAAAAGTGAACG-3′)	rbcL1444-(5′-GCGAAATCAGCTGTATCTGTWG-3′)	Ruck and Theriot, 2011
b	rbcL181F (5′-ACCGTGCTAAAGCTT-3′)	rbcL1174R (5′-ACCRATTGTACCACC-3′)	This study
a	18SP2F (5′-CTGGTTGATTCTGCCAGT-3′)	18SP4R (5′-TGATCCTTCYGCAGGTTCAC-3′)	van Hannen et al., 1999; Guillou et al., 1999
b	18S200F (5′-YGGSRWGAYRTGGTGADTCA-3′)	18S1444R (5′-GVRTRCATCAGTGTAGCGCG-3′)	This study
a	psbAF (5′-ATGACTGCTACTTTAGAAAGACG-3′)	psbAR (5′-GCTAAATCTARWGGGAAGTTGTG-3′)	Yoon et al., 2002
b	psbA45Fdg (5′-WTTYATCGWGCTCCWCCAG-3)	psbA648R (5′-RTGWGCTGCAACGATRTTRT-3′)	This study

*Nested, a, refers to the first primer amplifications; b, the primer used in the second amplification*.

The first amplifications were performed in a 25 μL volume with a final concentration of 1 × PCR buffer (Bioshop Canada Inc.), 2 mmol L^−1^ MgCl_2_, 0.3 mmol L^−1^ dNTP, 0.4 μmol L^−1^ of each primer, 1 unit of *Taq* DNA polymerase (BioShop), and 5.0 μl of Chelex DNA extract supernatant. The following cycling conditions were used: 94°C for 210 s; followed by 36 cycles of 94°C for 50 s, 52°C for 50 s, and 72°C for 80 s; and then a final elongation step at 72°C for 15 min. For the second amplification (and the third amplification step when performed), all steps and concentrations were the same as above except that 1 μl of the product from the previous amplification was used as template. The success of the PCR was assessed by visualizing the products on a 1.5% agarose gel. Successful PCR products were purified using the enzymes Exonuclease I and shrimp alkaline phosphatase (USB Corporation). Big Dye version 3.1 (Life Technologies Corporation) was used for sequencing reactions using 0.6 μL of Big Dye in a 10 μL reaction. Sequencing reaction products were purified via ethanol-EDTA-sodium acetate precipitation. Nucleotide sequences were generated using automated cycle-sequencing on an Applied Biosystems 3130xl automated sequencer. To validate the use of Bioshop Taq DNA polymerase, seven of the samples were re-amplified for all three genes using Phusion® High-Fidelity DNA Polymerase (New England Biolabs). The optimized annealing temperatures used in the first and second Phusion amplifications were as follows: rbcL 57.7°C and 60.3°C; 18S 52.2°C and 60.3°C; psbA 52.2°C and 63°C. Further, to ensure that the error rate was not affecting our sequences, seven samples were re-amplified for rbcL using Phusion with 20, 25, and 30 cycles in the second amplification. The final PCR products were then sequenced and compared for base pair differences with the sequences obtained with Bioshop Taq using the standard 34 cycle second amplification step.

Seventy-two rbcL sequences were studied from a wide range of diatom taxa. Within the pennate raphe bearing diatoms, 35 diatom cells were sequenced for all three genes to ensure that the method would allow for multi-gene sequencing. Sequences were assembled and edited in Geneious version 6.1.5 and consensus sequences were aligned using the MAFFT alignment tool. Consensus sequences were compared to the GenBank database using the Basic Local Alignment Search Tool (BLAST) to verify and ensure that no contaminants were sequenced. Initial Maximum Parsimony (MP) tree topologies of each gene were assessed in PAUP v.4.0 (Swofford, 2003), and phylogenetic model testing (using likelihood scores and AIC calculations) of each region was analyzed in JModel Test v.2.1.4 (Guindon and Gascuel, 2003; Darriba et al., 2012) to ensure that the data could be concatenated for analysis. Datasets had the preferred General Time Reversible model (GTR+I+G) (Tavaré, 1986) except the 18S dataset which had the Transitional Model (TIM3+I+G) (Posada, 2003). However, the initial topology tree of the 18S matched both the rbcL and psbA initial topology trees, and, using a chi-squared distribution, the delta values from TIM3+I+G and GTR+I+G (delta = 0.0000, *K* = 78; delta = 3.9436, *K* = 80, respectively) were not shown to be statistically different (*P* < 0.15). Therefore, the model GTR+I+G was used for Bayesian analysis (BI) and Maximum Likelihood (ML) for both the concatenated data set (rbcL, 18S, psbA) and the single gene dataset (rbcL). The BI was carried out with MrBayes v.3.1.2 (Huelsenbeck and Ronquist, 2001; Ronquist and Huelsenbeck, 2003), with a Monte Carlo Markov Chain (MCMC) run for 1 million generations for the concatenated gene data set and 5 million generations for the rbcL dataset with the default settings. Runs were sampled every 1000th generation. The first 250,000 and 1,250,000 were discarded as burn-in for the concatenated gene dataset and rbcL dataset, respectively. The convergence and stationarity of the BI results were analyzed in Tracer v1.6 (Rambaut et al., 2013) and topology convergences were analyzed in AWTY (Wilgenbusch et al., 2004). ML Bootstrap analysis (Felsenstein, 1985) was done in Garli v.2.01 (Zwickl, 2006), using the GTR+I+G model, with 1000 bootstrap replicates for both the concatenated dataset and rbcL data set. *Fragilaria bidens* (GenBank Acquisition AB430716.1) was used as the outgroup for our concatenated dataset as it was a close sister species to the taxa used in the concatenated analysis. *Bolidomonas pacifica* (GenBank Acquisition HQ912421.1) and *Cyclotella meneghiniana* (GenBank Acquisition KF959651.1) were used as outgroup and sister taxa, respectively, for the rbcL dataset as they were the closest relatives to the dataset taxa available on GenBank.

## Results

Partial sequences for rbcL (1202–1305 bp), 18S (811–1144 bp), and psbA (537–578 bp) were determined for 35 single cell freshwater diatom isolates. An additional 37 partial rbcL sequences were determined for a variety of diatom genera including *Melosira* C.Ag., *Aulacoseira* Thwaites*, Synedra* (*Ulnaria*) Ehrenberg, *Eunotia* Ehrenberg, *Navicula* Bory*, Neidium* Pfitzer, *Placoneis* C.Mereschkowsky, *Frustulia* C.Ag., *Gyrosigma* Hassall, *Stauroneis* Ehrenberg, *Craticula* Grunow, *Sellaphora* C.Mereschkowsky, *Pinnularia* Ehrenberg, *Cymatopleura* W.Sm., *Encyonema* Kütz., *Gomphonema* Ehrenberg, *Nitzschia* Hassall, *Hantzschia* Grunow, and *Surirella* Turpin.

Of the diatoms sequenced, 60 were from fresh living samples, 12 were from RNA*later* fixed samples, and one from a Lugol's fixed sample (Tables 1, 2, Supplement 1 in Appendix). NCBI Blast searches using the new sequences resulted in matches consistent with the genus-level morphological identifications of our specimens. Method validation of the number of cycles (20, 25, 30, and 34) and type of DNA polymerase using five different taxa showed only one instance of base pair substitutions, though there we no differences in the overall sequence alignments. The recovered sequence lengths for rbcL and 18S were both within the average range for the diatom sequences found on GenBank (Table 4). The length of psbA sequence recovered was slightly below the gene length found for diatom sequences on Genbank (Table 4). The amplification success was 70%, 90%, and 96% for rbcL, 18S, and psbA, respectively (Table 5). The recovery success of 18S and psbA was higher than rbcL because only samples that amplified successfully for rbcL were processed for these two regions. We had very low amplification success with the Lugol's fixed samples (Table 5). In addition, within the Lugol's fixed samples 13 contained fungi 18S nuclear DNA. RNA*later* amplification success was consistent across sample storage periods ranging from 5 to 21 days (Table 5).

**Table 4:** **Range of sequence base pair lengths for the three genes studied from our samples and those reported from GenBank**.

	**Our samples**	**GenBank samples**	**Mean**
*rbcL*	1202–1298	364–1475	1181 (±386)
*18S*	996–1145	3–1915	818 (±556)
*psbA*	537–578	660–1082	790 (±110)

*Mean ± SD represents base pair lengths from GenBank sequences*.

**Table 5:** **Amplification success rates for live, RNA*later*, and Lugol's solution samples**.

**Gene**	**Live**	**RNAlater**				**Lugol's**
		**5–7 days storage**	**8–14 days storage**	**15–21 days storage**	**22–30 days storage**	
	**Successful (total attempts)**	**Successful (total attempts)**	**Successful (total attempts)**	**Successful (total attempts)**	**Successful (total attempts)**	**Successful (total attempts)**
*rbcL*	348 (446)	9 (15)	1 (3)	4 (13)	4 (7)	4 (53)
*18S*	183 (201)	7 (8)	1 (1)	3 (4)	1 (3)	13^*^ (19)
*psbA*	65 (66)	7 (7)	1 (1)	4 (4)	NA	1 (1)

* *All of the successful samples were found to contain residual fungi DNA*.

### Multiple gene analysis

**Figure 1:**
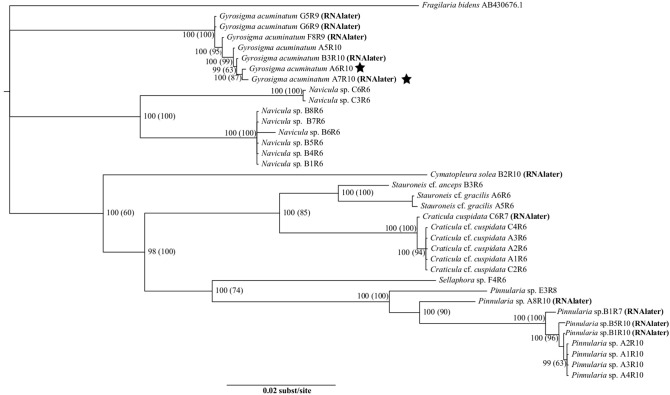
**Tree showing relationships of rbcL, 18S, and psbA using the best fit model, GTR+I+G**. Statistical support is shown with numbers at nodes: Bayesian posterior probabilities (Maximum Likelihood bootstrap values). The stars indicates two *Gyrosigma* cells, one from a fresh sample and the other from an RNAlater preserved samples which had significantly similar sequences. Taxa are indicated to be from either RNA*later* or fresh samples.

Individual topologies of the three genes (rbcL, 18S, psbA) showed no differences, neither did the ML (-LnL = 10058.4412) nor BI analyses, thus only the BI tree was shown with both the BI posterior probabilities (PP) and the ML bootstrap values (BS) (Figure 1). Our dataset showed significant separation at the family level for the following: Pinnulariaceae (PP = 100, BS = 100), Sellaphoraceae (100, 74), Stauroneidaceae (100, 85), Pleurosigmataceae (100, 100), Naviculaceae (100, 100). RNA*later* preserved and fresh samples of the same taxa were found within the correct clades. Examples of this can be seen in the genera *Craticula, Gyrosigma*, and *Pinnularia* (Figure 1). In the genus *Gyrosigma*, a fresh sample and an RNA*later* sample were significantly similar (100, 87), and came out on the same terminal branch (Figure 1, stars). The small branching of individual taxa within this genera were due to ≤5 bp differences. Although difficult to determine, low number of base pair differences could be either base pair substitution error or intrageneric variation (0.001%) between the concatenated sequences (>3000 bp).

Slight branching between individuals was also present in the genera of *Craticula* (≤ 3 bp differences, 0.001%) and *Pinnularia* (≤ 14 bp differences, 0.004%), showing low levels of intrageneric variation (Figure 1). Individual cells were principally collected from benthic sediments, leading to the larger representation of Naviculaceae taxa.

### Single gene analysis

**Figure 2:**
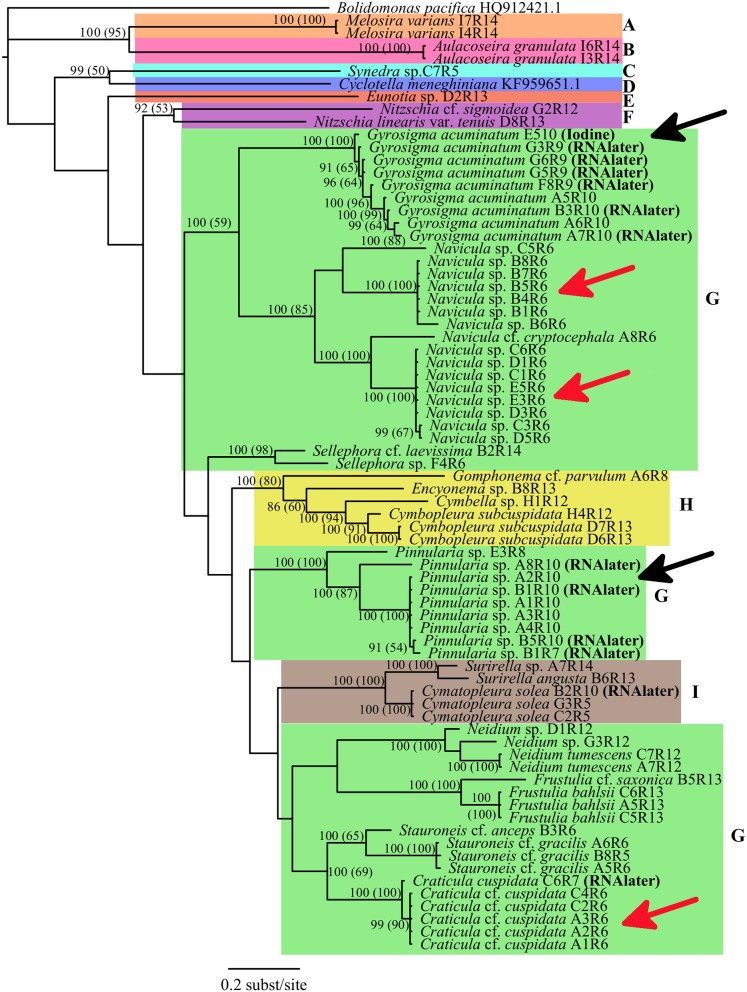
**Phylogenetic relationships of Bacillariophyta rooted with ***Bolidomonas pacifica*** based on a rbcLdataset using best fit model GTR+I+G with BI and MLBS anaylses**. Numbers at the nodes indicate statistical support if both methods resulted in >50%: Bayesian Inference posterior probabilities (Maximum Likelihood bootstrap values). Taxa are indicated to be from either fresh, RNALater or iodine samples. Color blocks indicate the different orders of Bacillariophyta (A, Melosirales; B, Aulacoseirales;C, Fragilariales; D, Thalassiosirales; E, Eunotiales; F, Bacillariales; G, Naviculales; H, Cymbellales; I, Surirellales). The black arrows show two instances in which sequences from preserved cells had very similar sequences from fresh cell samples. The red arrows show three instances where taxa collected from the same location had identical sequences. *Bolidomonas pacifica* (GenBank Acquisition HQ912421.1) and *Cyclotella meneghiniana* (GenBank Acquisition KF959651.1) were used as an outgroup and sister group respectively, and their rbcL sequences were obtained from GenBank.

For the single gene dataset using only rbcL, neither the ML (-LnL = 9690.8210) nor the BI trees showed any differences, thus only the BI tree was shown with both the BI PP and the ML BS values (Figure 2). All isolates from the same genus showed strongly supported monophyletic taxa. In particular, the genera *Craticula, Pinnularia, Neidium, Frustulia, Cymatopleura, Surirella, Gyrosigma, Melosira*, and *Aulocoseira* all had very high support values (PP = 100; BS = 100), while the genera *Stauroneis* (100, 65) and *Navicula* (100, 85) had supported monophyletic taxa groups. Cells of the same taxon, collected from the same location (±10 m area) were also more closely aligned in the tree compared to similar cell isolates of the same taxon from other locales (Figure 2, red arrows). Specimens from the same genus which were isolated from either fresh, RNA*later* preserved or in one case Lugol's preserved samples were always in the same monophyletic group. The *Gyrosigma* specimen which was isolated from iodine fixation was placed with all other *Gyrosigma* isolates (PP = 100, BS = 100). As well, for both *Gyrosigma* and *Pinnularia*, fresh and RNA*later* preserved isolates were on the same terminal branch (Figure 2, black arrows).

### Specimen and sample fixation

**Figure 3:**
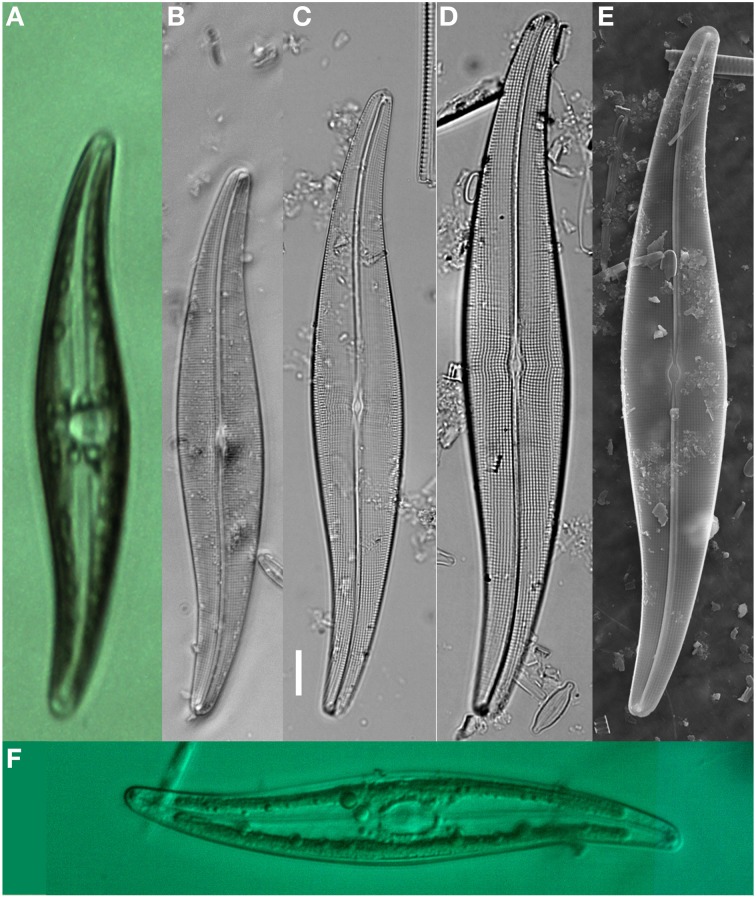
*****Gyrosigma acuminatum*** shown as live, fixed and cleaned specimens with light microscopy (LM) and scanning Electron microscopy (SEM)**. **(A)** Live specimen from NHC pond (sequenced for rbcL), low magnification showing valve outline, and cytoplasmic structure. **(B–D)** LM micrographs showing the size reduction series from one sample location. **(E)** SEM illustration the internal view of the valve. **(F)** LM image of an RNA*later* fixed specimen after freezing at −20°C and thawing. Scale bar = 10 μm.

Diatom specimens or populations for morphological study in association with DNA were cleaned and mounted for light microscopy (LM) and scanning electron microscopy (SEM) validation. A population of *Gyrosigma acuminatum* (Kütz.) Rabenh. for example (*n* = 30) illustrated a natural size diminution series (Length, 106.5–163.5; width, 18.5–27.5; stria density, 17–19 μm; areola density, 17–18 in 10 μm, Figures 3A–E). Additional specimens fixed in RNA*later* and frozen (−17°C) for 2 days maintained frustule integrity with cytoplasm and chloroplast structure (Figure 3F). Chloroplasts were intact however alterations in the structure were observed; in some specimens there was slight shrinkage apically and transapically, while others had poorly defined chloroplast walls. Cyanophyceae (e.g., *Phormidium* sp., *Oscillatoria* cf. *princeps* Vaucher ex Gomont) and Chlorophyceae [e.g., *Oedigonium* sp., *Pediastrum boryanum* (Turp.) Menegh.] cells also maintained cell structure. The Chlorophyceae had intact but sometimes slightly altered chloroplasts (pers. obs.)

## Discussion

The development of novel DNA extraction protocols has accelerated the exploitation of microbial genetic studies in health (e.g., Richlen and Barber, 2005), environment (e.g., Neilan, 1995; Kermarrec et al., 2013), and even diatom taxonomic research (e.g., Evans et al., 2007; Pouličková et al., 2010). Nested DNA amplifications have the potential to open the genetic vault of taxonomic information from single microbial cells. The simple methodology of single cell Chelex® DNA extraction followed by nested PCR has great implications for expanding the genetic reference library of information in algal research. This study uses diatoms as test organisms; preliminary PCR success using dinoflagellates (pers. obs.) is also recorded. The 70–96% amplification success rates using live and (31–100%) using RNA*later* fixed samples for single cell PCRs is similar to recovery rates using cultures (Lang and Kaczmarska, 2011). DNA polymerases of different quality and price points (BioShop Taq® and Phusion®) produced (93%) the same sequence results. Comparisons of sequences using NCBI BLAST also supported the morphological taxa identifications of the specimens. In this study, the systematic associations of *Gyrosigma* to *Navicula, Craticula* to *Stauroneis*, and *Pinnularia* to *Sellaphora* (Figures 1, 2) were in agreement with other studies (Evans et al., 2007; Theriot et al., 2010). This success rate increases the utility of conserved genes known to be good for species level taxonomic discriminations (Hamsher et al., 2011).

In this study, one existing nested primer set for rbcL was used while two new primer pairs were developed for the nested amplification of 18S and psbA in diatoms. The recovered sequence lengths for rbcL and 18S were within the average range of the lengths of diatom sequences found on GenBank while psbA sequence lengths were slightly below the average gene length found for diatom sequences (Table 4). One single cell DNA extraction provided enough amplification template for multiple DNA amplifications making the approach compatible for robust multi-gene analyses. In this study, 10 amplifications from a single cell extract were successfully performed. Based upon the replicated amplifications done, a conservative estimate suggests there could be enough template for 30–35 PCR amplifications per extraction. There is even potential that nested amplifications can be used to generate large-scale genetic datasets using next generation sequencing protocols optimized for low concentration DNA templates (e.g., initial amplification using the Multiple Displacement Amplification reaction, (Lasken, 2007); but see (Ning et al., 2014) for a review of current challenges). Some research suggests that the Chelex extraction method, which also conserves proteins, may not be the most suitable for Multiple Displacement Amplification reactions. However, one study found that keeping proteins was not an issue for whole gene amplification (Lepere et al., 2011).

One concern related to using this technique is the potential for contamination during isolation of a single cell. Often diatom cells, like taxa in the genera *Asterionella* Hassall, *Tabellaria* Ehrenberg and *Surirella*, have epiphytes which may contaminate amplifications. In addition, single cells often have organic extracellular polymeric substances (EPS) which capture bacteria, fungi, and loose organics with remnant DNA (Das et al., 2013). Problems related to the amplification of DNA from non-target sources were observed in this study. In the Lugol's fixed samples, 18S nuclear DNA recovery from fungi was amplified in 13 samples. In this instance, the contamination problem was identified by BLASTing the sequencing product. Contamination from non-algae sources can be easily identified using this protocol and removed from study. Since contamination was only observed in selected Lugol's samples, we can conclude that with good isolations, fungi can be effectively removed from sample concerns using fresh and freshly fixed material. Contamination from more genetically associated algal sources maybe more problematic given the potential for cross-contamination during amplifications (Zhang et al., 1992; Ruck et al., 2014) and a limited genetic reference library available for comparison. However, in this study there was no evidence of contamination.

Time consuming recovery of DNA from micro-organism cultures has limited the success of barcoding in the Protista. Ruck and Theriot (2011) developed a single cell diatom field isolation and rbcL DNA extraction procedure using Chelex. This approach is effective, although limited by time requirements for isolating cells in the field and no ability to reinvestigate the samples collected. Collecting live and fixed samples in the field for subsequent isolation back in the lab gives greater success in DNA recovery. Using this single cell extraction protocol alone, or with a limited number of replicated daughter cells (short-term cultures), will greatly increase the database of DNA sequences for diatoms and microbes.

**Figure A1:**
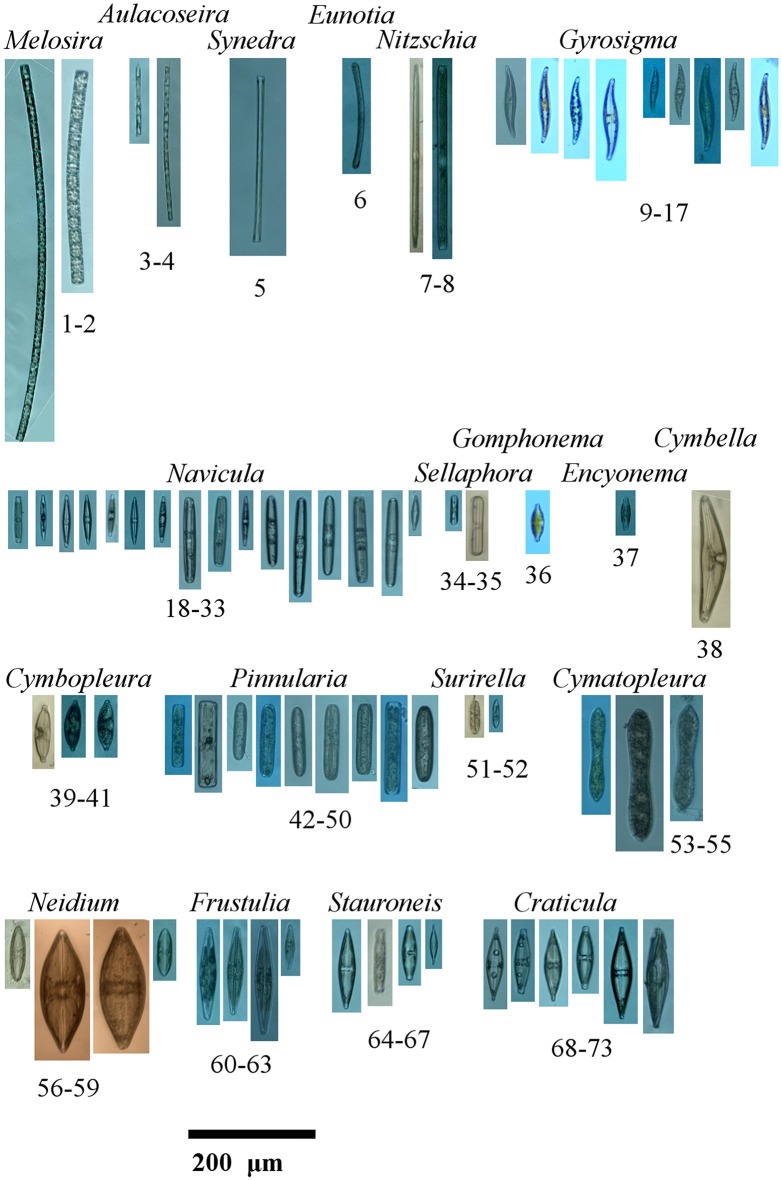
**Isolation photos of sequenced taxa aligned to their placement in Figure 2**. *Bolidomonas pacifica* (GenBank Acquisition HQ912421.1) and *Cyclotella meneghiniana* (GenBank Acquisition KF959651.1) are not pictured as their sequences were used as an out group and sister group from GenBank respectively. The isolated taxa are aligned as in Figure 2 and shown with names and identifier. 1, *Melosira varians* I7R14; 2, *Melosira varians* I4R14; 3, *Aulacoseira granulata* I6R14; 4, *Aulacoseira granulata* I3R14; 5, *Synedra* sp. C7R5; 6, *Eunotia* sp. D2R13; 7, *Nitschia* cf. *sigmoidea* G2R12; 8, *Nitschia linearis* var. *tenuis* D8R13; 9, *Gyrosigma acuminatum* E5R10; 10, *Gyrosigma acuminatum* G3R9; 11, *Gyrosigma acuminatum* G6R9; 12, *Gyrosigma acuminatum* G5R9; 13, *Gyrosigma acuminatum* F8R9; 14, *Gyrosigma acuminatum* A5R10; 15, *Gyrosigma acuminatum* B3R10; 16, *Gyrosigma acuminatum* A6R10; 17, *Gyrosigma acuminatum* A7R10; 18, *Navicula* sp. C5R6; 19, *Navicula* sp. B8R6; 20, *Navicula* sp. B7R6; 21, *Navicula* sp. B5R6; 22, *Navicula* sp. B4R6; 23, *Navicula* sp. B1R6; 24, *Navicula* sp. B6R6; 25, *Navicula* cf. *cryptocephala* A8R6; 26, *Navicula* sp. C6R6; 27, *Navicula* sp. D1R6; 28, *Navicula* sp. C1R6; 29, *Navicula* sp. E5R6; 30, *Navicula* sp. E3R6; 31, *Navicula* sp. D3R6; 32, *Navicula* sp. C3R6; 33, *Navicula* sp. D5R6; 34, *Sellaphora* sp. F6R4; 35, *Sellaphora* cf. *laevissima* B2R14; 36, *Gomphonema* cf. *parvulum* A6R8; 37, *Encyonema* sp. B8R13; 38, *Cymbella* sp. H1R12; 39, *Cymbopleura subcuspidata* H4R12; 40, *Cymbopleura subcuspidata* D7R13; 41, *Cymbopleura subcuspidata* D6R13; 42, *Pinnularia* sp. E3R8; 43, *Pinnularia* sp. A8R10; 44, *Pinnularia* sp. A2R10; 45, *Pinnularia* sp. B1R10; 46, *Pinnularia* sp. A1R10; 47, *Pinnularia* sp. A3R10; 48, *Pinnularia* sp. A4R10; 49, *Pinnularia* sp. B5R10; 50, *Pinnularia* sp. B1R7; 51, *Surirella* sp. A7R14; 52, *Surirella angusta* B6R13; 53, *Cymatopleura solea* B2R10; 54, *Cymatopleura solea* G3R5; 55, *Cymatopleura solea* C2R5; 56, *Neidium* sp. G3R12; 57, *Neidium tumescens* C7R12; 58, *Neidium tumescens* A7R12; 59, *Neidium* sp. D1R12; 60, *Frustulia bahlsii* C6R13; 61, *Frustulia bahlsii* A5R13; 62, *Frustulia bahlsii* C5R13; 63, *Frustulia saxonica* B5R13; 64, *Stauroneis* cf. *gracilis* A6R6; 65, *Stauroneis* cf. *gracilis* B8R5; 66, *Stauroneis* cf. *gracilis* A5R6; 67, *Stauroneis* cf. *anceps* B3R6; 68, *Craticula* cf. *cuspidata* C4R6; 69, *Craticula* cf. *cuspidata* C2R6; 70, *Craticula* cf. *cuspidata* A3R6; 71, *Craticula* cf. *cuspidata* A2R6; 72, *Craticula* cf. *cuspidata* A1R6; 73, *Craticula cuspidata* C6R7.

In cell sequencing, there are inherent problems with the destruction of the voucher specimen (Figure A1). Although reported that diatom valves can be recovered after DNA extraction in ethanol (Lang and Kaczmarska, 2011), the glass beads in the Chelex solution destroy diatom valves after centrifugation (pers. obs.). To limit the possibility of erroneous specimen identifications, many photomicrographs were taken of each single cell prior to DNA extraction. These were linked to morphological vouchers (cleaned diatom valves) collected from the same respective population. For example, diatom specimens of *G. acuminatum* were matched using DNA results and recoverable specimens from the natural population (Figure 3, Figure A1). To further reduce identification errors, replication of DNA sequencing results with comparison to more morphological specimens from a population can improve the validation of species identifications in association with both genetic and morphological variability. In the case of *G*. *acuminatum*, these gene sequences can be directly related to a morphological study of the species type (Sterrenberg, 1995). However, this approach can be highly problematic when taxa within a community have overlapping morphological characteristics. In these cases, additional detailed multi-gene studies of populations could enhance the resolution and identification of cryptic species (e.g., Pouličková et al., 2010).

Traditional fixation of microbial samples for morphological, ecological and physiological study has a long history (Throndsen, 1978; Simmons, 2014). Recent studies have demonstrated that gene sequences can be recovered from a variety of traditionally fixed samples (Connell, 2002; Godhe et al., 2002; Henrichs et al., 2007; Lang and Kaczmarska, 2011). Ethanol fixed samples are not commonly found in microbial museum collections because they are subject to evaporation and have negative extraction effects on cell pigments. In the current study, recovery of gene product from Lugol's fixed samples was poor 7.5 and 68% for rbcL and 18S genes respectively. With these product recoveries sequence success was reduced further with many isolates have low quality sequence results which were rejected. Only one psbC amplification and sequence determination was attempted and successful. No success was observed from formalin-fixed samples, although we did not immediately transfer formalin-fixed material into methanol storage as described by Godhe et al. (2002). Although Bertozzini et al. (2005), and Auinger et al. (2008) have successfully recovered DNA from dinoflagellates and chrysophyte fixed with Lugol's solution, we need more detailed methodological studies to improve percent success in the routine recover of DNA from diatoms in Lugol's and formalin fixation. However, Godhe et al. (2002) suggest that Lugol's solution and varying ethanol fixations have other shortcomings. In this study we also noted the presence of fungi in museum collections fixed with Lugol's, a potential problem with historically fixed collections. At present RNA*later* represents an excellent genetic fixation protocol for sample collection, short term storage and long-term archiving of microbial collections. Cell wall structure in Chlorophyceae (*Oedigonium* sp., *Pediastrum boryanum*), Cyanophyceae (*Phormidium* sp., *Oscillatoria* cf. *princeps*) and diatoms were maintained in RNA*later* fixed samples under cold and frozen conditions. Chloroplast integrity was even maintained for *G*. *acuminatum* during freezing at −17°C (Figure 3F). The specifications for RNA*later* indicate that treated tissues can be stored at 25°C for 1 week, at 4°C for 1 month, or at −20°C indefinitely (Ambion, 1999). In this study, both fresh and RNAlater fixed samples had predictable extraction success (Table 5). This supports the adoption of RNAlater as a long-term diatom storage media.

The recovery of DNA from archived museum and research collections is currently poor but quickly advancing, especially with vertebrate collections (e.g., Payne and Sorenson, 2002). However, museums and large collections should prioritize the implementation of storage and fixation techniques that maintain the molecular integrity of the samples. RNA*later* preserved algae, including diatoms, subjected to freeze-thaw cycles showed some internal cell cytoplasmic alterations; however the chloroplasts and associated pyrenoids remained intact. RNA*later* represents a good alternative for specimen, tissue and single cell preservation. DNA barcoding can help with species delimitation and refining the concept of cryptic species. For example in this rudimentary study with a small population of *G. acuminatum* (Figures 1, 3), gene sequences for rbcL showed 1–5 base pairs differences between the four specimen clades, collected from three different sites within our primary pond (NHC-1). *C*. cf. *cuspidata* showed no variability (no base pair differences) in specimens from another sample site. In contrast, up to 117 bp differences were observed within the *Navicula* clade from three different locations within a lake and up to 88 bp differences were noted in the *Pinnularia* clade from four different lakes and pond locations. These results suggest that by expanding the use of barcodes to many individuals within a diatom population, inter- and intraspecific questions can be routinely addressed.

Historical problems in extracting, amplifying, and sequencing DNA from single-cells have limited the development of genetics as a tool in the study of global microbial diversity, biogeography, and physiology. In diatoms, DNA sequencing from single cells is a logical step forward in population, taxonomy and environmental genetic studies. More conventional morphometric studies routinely use sample populations to determine size diminution series and variability of morphological expression (e.g., Lange-Bertalot et al., 2011; Levkov et al., 2013). With detailed genetic studies of single cells, links to match morphological populations will be informative in understanding variations in genotypic and phenotypic expression. At the species level, single cell multi-gene sequences along with associated morphometrics can act as multi-proxy validation datasets for species identifications. Future developments with single cell sequencing may even advance next generation genomic research.

## Author contributions

PH funded the project, developed in collaboration with RB the experimental design, identified the development with fixed samples, initiated the expansion to multiple gene and extractions, completed cell isolations and wrote the drafts of the manuscript. KL conducted cell isolations and the majority of the sequencing, edited and corrected the manuscript. This author developed new ideas on recovery of viable DNA, produced the figures and tables, wrote most of the methods and results. RB conducted the initial work in developing the single cell isolation protocol for the Canadian Museum of Nature Laboratory of Molecular Biodiversity, developed new primers for the nested amplification, experimental ideas for the development of the manuscript and contributed extensively to the writing and final editing of the manuscript.

### Conflict of interest statement

The authors declare that the research was conducted in the absence of any commercial or financial relationships that could be construed as a potential conflict of interest.

## Appendix

### Data accessibility

DNA sequence: Genbank accessions KM999080-KM999117. Final DNA sequence assembly: online support information.

Supplement 1: Light microscope images of the single cells used in the DNA sequencing, in Figures 1, 2.

Supplement 2: All original samples (CANA_100043-100099) are housed in the National Algae Collection of Canada (CANA), http://www.nature-cana.ca/databases/index.php.

## References

[B1] Alverson A. J. Jansen R. K. Theriot E. C. (2007). Bridging the rubicon: phylogenetic analysis reveals repeated colonizations of marine and fresh waters by thalassiosiroid diatoms. Mol. Phylogenet. Evol. 45, 193–210. 10.1016/j.ympev.2007.03.024 17553708

[B2] Amato A. Kooistra W. H. C. F. Ghiron J. H. L. Mann D. G. Pröschold T. Montresor M. (2007). Reproductive isolation among sympatric cryptic species in marine diatoms. Protist 158, 193–207. 10.1016/j.protis.2006.10.001 17145201

[B3] Ambion (1999). Preserve RNA and tissue cell samples with RNAlater®. Ambion TechNotes Newsl. 5, 7–8.

[B4] Auinger B. M. Pfandl K. Boenigk J. (2008). Improved methodology for identification of protists and microalgae from plankton samples preserved in Lugol's iodine solution: combining microscopic analysis with single-cell PCR. App. Environ. Microbiol. 74, 2505–2510. 10.1128/AEM.01803-07 18296536 PMC2293146

[B5] Bennett M. S. Wiegert K. E. Triemer R. E. (2014). Characterization of *Euglenaformis* gen. nov. and the chloroplast genome of Euglenaformis [Euglena] proxima (Euglenophyta). Phycologia 53, 66–73. 10.2216/13-198.1

[B6] Bertozzini E. Penna A. Pierboni E. Bruce I. Magnani M. (2005). Development of new procedures for the isolation of phytoplankton DNA from fixed samples. J. App. Phycol. 17, 223–229. 10.1007/s10811-005-2130-5

[B7] Bowers H. A. Tengs T. Glsaglow H. B. Jr. Burkholder J. M. Rublee P. A. Oldach D. W. (2000). Development of real-time PCR assays for rapid detection of *Pfiesteria piscicida* and related dinoflagellates. Appl. Environ. Microbiol. 66, 4641–4648. 10.1128/AEM.66.11.4641-4648.2000 11055905 PMC92361

[B8] CBOL Plant Work Group. (2009). A DNA barcode for land plants. Proc. Natl. Acad. Sci. U.S.A. 106, 12794–12797. 10.1073/pnas.0905845106 19666622 PMC2722355

[B9] Connell L. (2002). Rapid identification of marine algae (*Raphidophyceae*) using three-primer PCR amplification of nuclear internal transcribed spaces (ITS) regions from fresh and archived material. Phycologia 14, 15–21. 10.2216/i0031-8884-41-1-15.1

[B10] Darriba D. Taboada G. L. Doallo R. Posada D. (2012). jModelTest2: more models, new heuristics and parallel computing. Nat. Methods 9, 772. 10.1038/nmeth.2109 22847109 PMC4594756

[B11] Das T. Sehar S. Manefield M. (2013). The roles of extracellular DNA in the structural integrity of extracellular polymeric substance and bacteria biofilm development. Environ. Microb. Rep. 5, 778–786. 10.1111/1758-2229.12085 24249286

[B12] Daugbjerg N. Andersen R. A. (1997). A molecular phylogeny of the heterokont algae based on analysis of chloroplast-encoded rbcL sequence data. J. Phycol. 33, 1031–1041. 10.1111/j.0022-3646.1997.01031.x

[B13] Edvardsen B. Eikrem W. Throndsen J. Sáez A. G. Probert I. Medlin L. K. (2011). Ribosomal DNA phylogenies and a morphological revision provide the basis for a revised taxonomy of the Prymnesiales (*Haptophyta*). Eur. J. Phycol. 46, 202–228. 10.1080/09670262.2011.594095

[B14] Estes A. Dute R. R. (1994). Valve abnormalities in diatom clones maintained in long-term culture. Diatom Res. 9, 149–258. 10.1080/0269249x.1994.9705305

[B15] Evans K. M. Wortley A. H. Mann D. G. (2007). An assessment of potential diatom “barcode” genes (cox1, rbcL, 18S and ITS rDNA) and their effectiveness in determining relationships in *Sellaphora* (Bacillariophyta). Protist 158, 349–364. 10.1016/j.protis.2007.04.001 17581782

[B16] Felsenstein J. (1985). Confidence limits on phylogenies: an approach using the bootstrap. Evolution 39, 783–791. 10.2307/2408678 28561359

[B17] Godhe A. Anderson D. M. Rehnstam-Holm A.-S. (2002). PCR amplification of microalgal DNA for sequencing and species identification: studies on fixatives and algal growth stages. Harmful Algae 1, 375–382. 10.1016/S1568-9883(02)00049-5

[B18] Guillou L. Chrétiennot-Dinet M.-J. Medlin L. K. Claustre H. Loiseaux-Goër S. Vaulot D. (1999). *Bolidomonas*: a new genus with two species belonging to a new algal class, the Bolidophyceae (Heterokonta). J. Phycol. 35, 368–381. 10.1046/j.1529-8817.1999.3520368.x

[B19] Guindon S. Gascuel O. (2003). A simple, fast, and accurate algorithm to estimate large phylogenies by maximum-likelihood. Syst. Biol. 52, 696–704. 10.1080/10635150390235520 14530136

[B20] Hahn S. Zhong X. Y. Troeger C. Burgemeister R. Gloning K. Holzgreve W. (2000). Current applications of single-cell PCR. Cell Mol. Life Sci. 57, 96–105. 10.1007/s000180050501 10949583 PMC11147117

[B21] Hamsher S. E. Evans K. M. Mann D. G. Pouličková A. Saunders G. W. (2011). Barcoding diatoms: exploring alternatives to COI- 5P. Protist 162, 405–422. 10.1016/j.protis.2010.09.005 21239228

[B22] Hebert P. D. N. Cywinska A. Ball S. L. deWaard J. R. (2003). Biological identifications through DNA barcodes. Proc. Biol. Sci. 270, 313–321. 10.1098/rspb.2002.2218 12614582 PMC1691236

[B23] Hebert P. D. N. Penton E. H. Burns J. M. Janzen D. H. Hallwachs W. (2004). Ten species in one: DNA barcoding reveals cryptic species in neotropical sipper butterfly *Astraptes fulgerator*. Proc. Natl. Acad. Sci. U.S.A. 101, 14812–14817. 10.1073/pnas.0406166101 15465915 PMC522015

[B24] Henrichs D. W. Renshaw M. A. Santamaria C. A. Richardson B. Gold J. R. Campbell L. (2007). PCR amplification of microsatellites from single cells of *Karenia brevis* preserved in lugol's iodine solution. Mar. Biotechnol. 10, 122–127. 10.1007/s10126-007-9044-y 17922226

[B25] Huelsenbeck J. P. Ronquist F. (2001). MRBAYES: Bayesian inference of phylogenetic trees. Bioinformatics 17, 754–755. 10.1093/bioinformatics/17.8.754 11524383

[B26] Kaczmarska I. Mather L. Luddington I. A. Muise F. Ehrman J. M. (2014). Cryptic diversity in a cosmopolitan diatom known as *Asterionellopsis glacialis* (Fragilariaceae): implications for ecology, biogeography, and taxonomy. Am. J. Bot. 101, 267–286. 10.3732/ajb.1300306 24509794

[B27] Kermarrec L. Franc A. Rimet F. Chaumeil P. Humbert J. F. Bouchez A. (2013). Next-generation sequencing to inventory taxonomic diversity in eukaryotic communities: a test for freshwater diatoms. Mol. Ecol. Res. 13, 607–619. 10.1111/1755-0998.12105 23590277

[B28] Lang I. Kaczmarska I. (2011). A protocol for a single-cell PCR of diatoms from fixed samples: method validation using *Ditylum brightwellii* (T. West) Grunow. Diatom Res. 26, 43–49. 10.1080/0269249X.2011.573703

[B29] Lange-Bertalot H. Bak M. Witkowski A. (2011). Diatoms of the European inland waters and comparable habitats: Eunotia and some related taxa in Diatoms of Europe, Vol. 6, ed Lange-Bertalot H. (Koenigstein: Koeltz Scientific Books), 1–747.

[B30] Lasken R. S. (2007). Single-cell genomic sequencing using multiple displacement amplification. Curr. Opin. Microbiol. 10, 510–516. 10.1016/j.mib.2007.08.005 17923430

[B31] Legrand B. Mazancourt P. d. Durigon M. Khalifat V. Crainic K. (2002). DNA genotyping of unbuffered formalin fixed paraffin embedded tissues. Forensic Sci. Int. 125, 205–211. 10.1016/S0379-0738(01)00641-7 11909665

[B32] Lepere C. Demura M. Kawachi M. Romac S. Probert I. Vaulot D. (2011). Whole-genome amplification (WGA) of marine photosynthetic eukaryote populations. Microbiol. Ecol. 76, 513–523. 10.1111/j.1574-6941.2011.01072.x 21348885

[B33] Levkov Z. Metzeltin D. Pavlov A. (2013). Diatoms of the European inland waters and comparable habitats: *Luticola* and *Luticolopsis*, in Diatoms of Europe, Vol. 7, ed Lange-Bertalot H. (Koenigstein: Koeltz Scientific Books), 1–697.

[B34] Mann D. G. Sato S. Tobajo R. Vanormelingen P. Souffreau C. (2010). DNA barcoding for species identification and discovery in diatoms. Cryptogam. Algol. 31, 557–577.

[B35] McCourt R. M. Karol K. G. Bell J. Helm-Bychowski K. M. Grajewska A. Wojciechowski M. F. (2000). Phylogeny of the conjugating green algae (*Zygnemophyceae*) based on rbcL sequences. J. Phycol. 36, 747–758. 10.1046/j.1529-8817.2000.99106.x 29542160

[B36] Neilan B. A. (1995). Identification and phylogenetics analysis of toxigenic cyanobacteria by multiplex randomly amplified polymorphic DNA PCR. Appl. Environ. Microbiol. 61, 2286–2291. 16535049 10.1128/aem.61.6.2286-2291.1995 PMC1388467

[B37] Ning L. Liu G. Li G. Hou Y. Tong Y. He J. (2014). Current challenges in the bioinformatics of single cell genomics. Front. Oncol. 4:7. 10.3389/fonc.2014.00007 24478987 PMC3902584

[B38] Payne R. B. Sorenson M. D. (2002). Museum collections as sources of genetic data. Bonn. Zool. Beitr. 51, 97–104.

[B39] Posada D. (2003). Using Modeltest and PAUP^*^ to select a model of nucleotide substitution, in Current Protocols in Bioinformatics, eds Baxevanis A. D. Davidson D. B. Page R. D. M. Petsko G. A. Stern L. D. Stormo G. D. (New York, NY: John Wiley and Sons), 6.5.1–6.5.14. 10.1002/0471250953.bi0605s00 18428705

[B40] Pouličková A. Veselá J. Neustupa J. Škaloud P. (2010). Pseudocryptic diversity versus cosmopolitanism in diatoms: a case study on *Navicula cryptocephala* Kütz (*Bacillariophyceae*) and morphologically similar taxa. Protist 161, 353–369. 10.1016/j.protis.2009.12.003 20097131

[B41] Rambaut A. Suchard M. A. Xie D. Drummond A. J. (2013). Tracer v1.5. Available online at: http://beast.bio.ed.ac.uk/Tracer

[B42] Reyes-Escogido L. Balam-Chi M. Rodríguez-Buenfil I. Valdés J. Kameyama L. Martínez-Pérez F. (2010). Purification of bacterial genomic DNA in less than 20 min using Chelex-100 microwave: examples from strains of lactic acid bacteria isolated from soil samples. Antonie Van Leeuwenhoek 98, 465–474. 10.1007/s10482-010-9462-0 20556655

[B43] Richlen M. L. Barber P. H. (2005). A technique for the rapid extraction of microalgal DNA from single live and preserved cells. Mol. Ecol. Notes 5, 688–691. 10.1111/j.1471-8286.2005.01032.x

[B44] Ronquist F. Huelsenbeck J. P. (2003). MrBayes 3: bayesian phylogenetic inference under mixed models. Bioinformatics 19, 1572–1574. 10.1093/bioinformatics/btg180 12912839

[B45] Ruck E. C. Nakov T. Jansen R. K. Theriot E. C. Alverson A. J. (2014). Serial gene losses and foreign DNA underlie size and sequence variation in the plastid genome of diatoms. Genome Biol. Evol. 6, 644–654. 10.1093/gbe/evu039 24567305 PMC3971590

[B46] Ruck E. C. Theriot E. C. (2011). Origin and evolution of the canal raphe system in diatoms. Protist 162, 723–737. 10.1016/j.protis.2011.02.003 21440497

[B47] Ruiz Sebastián C. O'Ryan C. (2001). Single-cell sequencing of dinoflagellate (*Dinophyceae*) nuclear ribosomal genes. Mol. Ecol. Notes 1, 329–331. 10.1046/j.1471-8278.2001.00084.x

[B48] Sherbakova T. A. Rubtsov N. B. Likhoshway Y. V. Grachev M. A. (2000). Combined SEM ultrstructure studies and PCR with individual diatom cells. Diatom Res. 15, 349–354. 10.1080/0269249X.2000.9705501

[B49] Simmons J. E. (2014). Fluid Preservation a Comprehensive Reference. Plymouth: Rowman and Littlefield.

[B50] Souffreau C. Verbruggen H. Wolfe A. P. Vanormelingen P. Siver P. A. Cox E. J.. (2011). A time-calibrated multi-gene phylogeny of the diatom genus *Pinnularia*. Mol. Phylogenet. Evol. 61, 866–879. 10.1016/j.ympev.2011.08.031 21930222

[B51] Sterrenberg F. A. S. (1995). Studies on the genus *Gyrosigma* and *Pleurosigma* (*Bacillariophyceae*): *Gyrosigma acuminatum* (Kützing) Rabenhorst, *G. spenceri* (Quekett) Griffith, and *G. rautenbachiae* Cholnoky. Proc. Acad. Nat. Sci. Phila. 146, 467–480.

[B52] Swofford D. L. (2003). PAUP^*^. Phylogenentic Analysis using Parsimony (^*^and other Methods). Version 4. Sunderland, MA: Sinauer Associates.

[B53] Tavaré S. (1986). Some probabilistic and statistical problems in the analyses of DNA sequences, in Some Mathematical Questions in Biology—DNA Sequence Analysis, ed Miura R. M. (Providence, RI: American Mathematical Society), 57–86.

[B54] Theriot E. C. Ashworth M. Ruck E. Nakov T. Jansen R. K. (2010). A preliminary multigene phylogeny of the diatoms (Bacillariophyta): challenges for future research. Plant Ecol. Evol. 143, 278–296. 10.5091/plecevo.2010.418

[B55] Throndsen J. (1978). Preservatives and storage, in Phytoplankton Manual. Monographs on Oceanographic Methodology 6, ed Sournia A. (Paris: United Nations Educational, Scientific and Cultural Organization), 69–74.

[B56] Tomitani A. Knoll A. H. Cavanaugh C. M. Ohno T. (2006). The evolutionary diversification of cyanobacteria: molecular-phylogenetic and paleontological perspectives. Proc. Natl. Acad. Sci. U.S.A. 103, 5442–5447. 10.1073/pnas.0600999103 16569695 PMC1459374

[B57] Trainor F. R. Rowland H. L. Lyllis J. C. Winter P. A. Bonanomi P. L. (1971). Some examples of polymorphism in algae. Phycologia 10, 113–119. 10.2216/i0031-8884-10-1-113.1

[B58] van Hannen E. J. Mooij W. van Agterveld M. P. Gons H. J. Laanbroek H. J. (1999). Detritus-dependent development of the microbial community in an experimental system: qualitative analysis by denaturing gradient gel electrophoresis. Appl. Environ. Microbiol. 65, 2478–2484. 10347030 10.1128/aem.65.6.2478-2484.1999 PMC91365

[B59] Vanormelingen P. Chepurnov V. A. Mann D. G. Sabbe K. Vyverman W. (2008). Genetic divergence and reproductive barriers among morphologically heterogeneous sympatric clones of *Eunotia bilunaris* sensu sato (Bacillariophyta). Protist 159, 73–90. 10.1016/j.protis.2007.08.004 17964215

[B60] Wilgenbusch J. C. Warren D. L. Swofford D. L. (2004). AWTY: A System for Graphical Exploration of MCMC Convergence in Bayesian Phylogenetic Inference. Available online at: http://ceb.csit.fsu.edu/awty 10.1093/bioinformatics/btm388 17766271

[B61] Yoon H. S. Hackett J. D. Pinto G. Bhattacharya D. (2002). The single, ancient origin of chromist plastids. Proc. Natl. Acad. Sci. U.S.A. 99, 15507–15512. 10.1073/pnas.242379899 12438651 PMC137747

[B62] Zhang L. Cui X. Schmitt K. Hubert R. Navidi W. Arnhein N. (1992). Whole genome amplifications from a single cell: implications for gene analysis. Proc. Natl. Acad. Sci. U.S.A. 89, 5847–5851. 10.1073/pnas.89.13.5847 1631067 PMC49394

[B63] Zimmermann J. Jahn R. Gemeinholzer B. (2011). Barcoding diatoms: evaluation of the V4 subregion on the 18S rRNA gene, including new primers and protocols. Org. Divers. Evol. 11, 173–192. 10.1007/s13127-011-0050-6

[B64] Zwickl D. J. (2006). Genetic Algorithm Approaches for the Phylogenetic Analysis of Large Biological Sequence Datasets under the Maximum Likelihood Criterion. Ph.D. dissertation, The University of Texas at Austin, USA, 1–115.

